# Physical activity level and its clinical correlates in chronic obstructive pulmonary disease: a cross-sectional study

**DOI:** 10.1186/1465-9921-14-128

**Published:** 2013-11-15

**Authors:** Mikael Andersson, Frode Slinde, Anne Marie Grönberg, Ulla Svantesson, Christer Janson, Margareta Emtner

**Affiliations:** 1 Department of Neuroscience, Physiotherapy, Uppsala University, Box 593/BMC, SE-751 24, Uppsala, Sweden; 2 Department of Medical Science, Respiratory Medicine and Allergology, Uppsala University, Uppsala, Sweden; 3 Department of Internal medicine and Clinical Nutrition, The Sahlgrenska Academy, University of Gothenburg, Gothenburg, Sweden; 4 Department of Clinical Neuroscience and Rehabilitation, The Sahlgrenska Academy, University of Gothenburg, Gothenburg, Sweden

**Keywords:** Physical activity, Activity monitor, COPD, Physical function, Body composition

## Abstract

**Background:**

Decreased physical activity is associated with higher mortality in subjects with COPD. The aim of this study was to assess clinical characteristics and physical activity levels (PALs) in subjects with COPD.

**Methods:**

Seventy-three subjects with COPD (67 ± 7 yrs, 44 female) with one-second forced expiratory volume percentage (FEV_1_%) predicted values of 43 ± 16 were included. The ratio of total energy expenditure (TEE) and resting metabolic rate (RMR) was used to define the physical activity level (PAL) (PAL = TEE/RMR). TEE was assessed with an activity monitor (ActiReg), and RMR was measured by indirect calorimetry. Walking speed (measured over 30-meters), maximal quadriceps muscle strength, fat-free mass and systemic inflammation were measured as clinical characteristics. Hierarchical linear regression was applied to investigate the explanatory values of the clinical correlates to PAL.

**Results:**

The mean PAL was 1.47 ± 0.19, and 92% of subjects were classified as physically very inactive or sedentary. The walking speed was 1.02 ± 0.23 m/s, the quadriceps strength was 31.3 ± 11.2 kg, and the fat-free mass index (FFMI) was 15.7 ± 2.3 kg/m^2^, identifying 42% of subjects as slow walkers, 21% as muscle-weak and 49% as FFM-depleted. The regression model explained 45.5% (p < 0.001) of the variance in PAL. The FEV_1_% predicted explained the largest proportion (22.5%), with further improvements in the model from walking speed (10.1%), muscle strength (7.0%) and FFMI (3.0%). Neither age, gender nor systemic inflammation contributed to the model.

**Conclusions:**

Apart from lung function, walking speed and muscle strength are important correlates of physical activity. Further explorations of the longitudinal effects of the factors characterizing the most inactive subjects are warranted.

## Background

Chronic obstructive pulmonary disease (COPD) is characterized by non-reversible airflow limitation and systemic effects [1]. In addition to respiratory impairments reduced exercise capacity, systemic inflammation, loss of muscle mass and reduced physical activity are common [2-6].

The impaired exercise capacity can be improved through pulmonary rehabilitation, but whether this increased functional reserve will lead to subjects utilizing their capacity in everyday life is still uncertain [7,8]. Both decreased exercise capacity and decreased physical activity are associated with higher mortality from COPD [9-11], indicating that further exploration of the factors involved in this complex interrelationship is warranted. If mediating factors are identified that contribute to variations in activity, they would be possible targets for interventions [12,13].

We hypothesized that using a combination of objectively measured clinical characteristics would be useful for assessing physical activity levels in subjects with COPD. Therefore, the aim of this study was to explore the clinical characteristics of physical activity in patients with moderate and severe COPD, with special emphasis on variables that are amendable through rehabilitation efforts.

## Methods

### Design and subjects

A cross-sectional study was performed, and a sample of 73 subjects with COPD was consecutively recruited from the Department of Respiratory Medicine, Sahlgrenska University Hospital, Gothenburg, Sweden. The inclusion criteria were: clinical diagnosis of COPD with a ratio of forced expiratory volume in one second (FEV_1_) by forced vital capacity of two standardized residuals below predicted value, smoking history ≥ 10 pack-years and stable disease. The exclusion criteria were: other conditions known to affect muscular tissue or physical performance (e.g., chronic heart failure, renal failure, rheumatic disease, diabetes, severe arthritis). The study was approved by the regional ethics review board in Gothenburg (Dnr: 408/05).

### Lung function

Post-bronchodilator lung function values from the subjects’ medical records were used if results from within six months were available; otherwise, dynamic spirometry was performed (SensorMedics model 922, SensorMedics Co., Palm Springs, CA, USA) [14]. Reference values from the European Community for Steel and Coal were used [15]. The severity of airflow limitation was based on post-bronchodilatory FEV_1_ using the criteria of Global Initiative for Chronic Obstructive Lung Disease (GOLD) [1].

### Energy expenditure

Resting metabolic rate (RMR) was measured by indirect calorimetry using a ventilated hood system (Deltatrac II Metabolic Monitor, Datex, Helsinki, Finland). Measurements were taken after an overnight fast, in a temperature-neutral environment with the subject in supine position and after 30 minutes of rest. The measurement period was 30 minutes, and the last 25 minutes were used for determining the RMR.

### Physical activity

The ActiReg activity monitor (Premed AS, Oslo, Norway) was used to measure physical activity [16]. The monitor consists of a storage unit (measuring 85×45×15 mm, weight 60 g) worn at the waist to which two pairs of sensors are attached: one pair at the front of the right thigh, the other at the chest. Each pair consists of a position sensor and a motion sensor. The position sensors discriminate between four body positions (lying, sitting, standing and bending forward) and the motion sensors between four states of motion (no motion, motion at chest or leg, and motion at both location). Information from the sensors are checked every second and by combining this information with the energy requirements of different body positions and activities [17], an estimate of total energy expenditure during the measurement period is possible in the software. Data were analyzed using the ActiCalc computer software (Premed AS, Oslo, Norway), with which total energy expenditure was analyzed and physical activity level calculated. The ActiReg system is validated for use in subjects with COPD and has shown high agreement with the doubly labeled water method for energy expenditure estimates [18]. The ActiReg was worn for seven consecutive days, except during the night or when taking a shower.

### Physical activity level

The physical activity level (PAL) was calculated as the ratio of the total energy expenditure (TEE) from the ActiReg divided by the RMR from indirect calorimetry (PAL = TEE/RMR). The World Health Organization proposes that the resulting ratio can be used to classify the lifestyle patterns of subjects as *very inactive* [PAL < 1.40], *sedentary or lightly active* [PAL 1.40 – 1.69], *active or moderately active* [PAL 1.70 – 1.99] or *vigorous or vigorously active* [PAL 2.00 – 2.40] [17].

### Walking speed

Walking speed was assessed by the 30-meter walk test [19]. The test was performed at two walking speeds, self-selected (usual) and maximal. Subjects were instructed to start from a standstill and walk 30 metres in both speeds with three minutes of rest in between. The mean walking speed at both the self-selected and the maximal speeds was calculated (distance/time, m/s). The cut-off for normal walking speed was set at 1.0 m/s [20].

### Quadriceps strength

Maximal voluntary isometric knee extensor strength was assessed using the Steve Strong dynamometer (SteveStrong HB, Gothenburg, Sweden). The outcome was maximal strength measured in Newtons (N). In a seated position with back support, hip and knee in 90 degrees, with the subject secured to the seat by a strap to minimize engagement of the muscles of the hip, three maximal efforts per leg were performed with vigorous encouragement. The highest result obtained from three maximal efforts was used. Data were converted into kg, and predicted values based on the equation by Seymour et al. [21] were used to classify muscle weakness.

### Anthropometrics and body composition

Height (m) and body weight (kg) was measured, and body mass index calculated as weight/height^2^ (kg/m^2^). Fat-free mass (FFM) was assessed in grams (g) by dual-energy x-ray absorptiometry (Lunar Prodigy, GE Healthcare, United Kingdom). The fat-free mass index (FFMI) was calculated as FFM/height^2^ (kg/m^2^). Fat-free mass depletion was defined as FFMI ≤ 15 for women and ≤ 16 for men [5].

### Systemic inflammation

Venous blood samples were taken in the morning after 12 hours of fasting. C-reactive protein (CRP) was used to assess systemic inflammation and analyzed according to standardized procedures at the Department of Clinical Chemistry, Sahlgrenska University Hospital, Gothenburg, Sweden.

### Dyspnea

Dyspnea during daily activities was assessed using the modified Medical Research Council scale, ranging from 0–4 [22].

## Statistics

The analyses were performed using IBM SPSS statistics version 20. Descriptive statistics are presented as the means (standard deviations) for continuous and normally distributed variables or as the medians (interquartile ranges) for categorical variables (or non-normally distributed variables). Skewed variables were log-transformed prior to analysis. Using PAL as a grouping variable, the sample was split into tertiles to explore whether a linear trend would be evident in the independent variables. Pearson product–moment correlation coefficient, *r* and Spearman rank correlation, *r*_*s*_ were used to assess associations. The Kruskal-Wallis test was used to test for differences in PALs between GOLD grades with the Mann–Whitney *U* test used for post hoc pairwise comparisons and gender differences.

Hierarchical multiple linear regression analysis was used to investigate the explanatory values of age, gender, FEV_1_% predicted, self-selected walk speed, quadriceps strength, FFMI and the log of CRP (independent variables) on PAL (dependent variable). In the case of missing values for independent variables, the multiple imputation technique was used to complete the data set. No variable had missing values in > 6% of cases. The significance level was set at p < .05. Due to the exploratory nature of the study no sample size calculation was performed.

## Results

Data from one subject’s activity monitoring were faulty and therefore excluded, leaving a total of 72 subjects (44 female) for analysis. The subjects’ mean age was 65 ± 7 years, and FEV_1_% predicted was 43 ± 16 (Table 1). Airway obstruction according to GOLD was grade 1 (n = 1), grade 2 (n = 18), grade 3 (n = 37) or grade 4 (n = 16).

**Table 1:** Description of sample. Numbers are means ± standard deviations, medians (IQRs) or numbers (%)

	**Total sample (n = 72)**	**1**^**st **^**tertile of PAL (n = 24)**	**2**^**nd **^**tertile of PAL (n = 24)**	**3**^**rd **^**tertile of PAL (n = 24)**
		**(1.15 – 1.38)**	**(1.40 – 1.51)**	**(1.52 – 2.21)**
Age, years	65 ± 7	65 ± 7	66 ± 7	65 ± 7
Gender, n male/female	28/44	8/16	10/14	10/14
Current smoker, n (%)	20 (28%)	7 (29%)	7 (29%)	6 (25%)
Dyspnea, mMRC	3 (1)	3 (1)	2.5 (2)	2 (2)
FEV_1_, percent of predicted	43 ± 16	35 ± 11	42 ± 16	52 ± 14
FVC, percent of predicted	83 ± 20	73 ± 19	85 ± 21	92 ± 20
GOLD grade 1/2/3/4, n	1/18/37/16	0/1/15/8	0/6/11/7	1/11/11/1
BMI, kg/m^2^	24.4 ± 4.7	23.2 ± 5.0	23.9 ± 4.0	26.0 ± 4.8
FFMI, kg/m^2^	15.7 ± 2.3	15.0 ± 2.4	15.5 ± 1.9	16.6 ± 2.3
FFM depleted, n (%)	35 (49%)	17 (71%)	12 (50%)	6 (25%)
Quadriceps strength, kg	31.3 ± 11.2	27.2 ± 10.7	30.2 ± 10.0	36.3 ± 11.3
Quadriceps strength, percent of predicted	81.8 ± 24.8	73.8 ± 28.0	80.8 ± 20.8	90.9 ± 23.1
Quadriceps weakness, n (%)	15 (21%)	6 (25%)	5 (21%)	4 (17%)
Walking speed, self-selected (m/s)	1.02 ± 0.23	0.89 ± 0.21	1.00 ± 0.23	1.14 ± 0.17
Walking speed, maximal (m/s)	1.54 ± 0.30	1.42 ± 0.29	1.57 ± 0.29	1.66 ± 0.30
Low self-selected walking speed, n (%)	30 (42%)	15 (63%)	11 (46%)	4 (17%)
CRP	1.86 ± 2.85	2.38 ± 3.24	1.47 ± 2.55	1.85 ± 2.75

mMRC = modified Medical Research Council scale (0-4), FEV_1_ = Forced expiratory volume in one second, FVC = Forced vital capacity, GOLD = Global Initiative for Chronic Obstructive Lung Disease, BMI = body mass index, FFM = fat-free mass, FFMI = fat-free mass index, FFM depleted = FFMI ≤ 15 in women, ≤16 in men, Slow walkers = < 1.0 m/s self-selected speed, CRP = C-reactive protein (anti-logged values).

**Figure 1:**
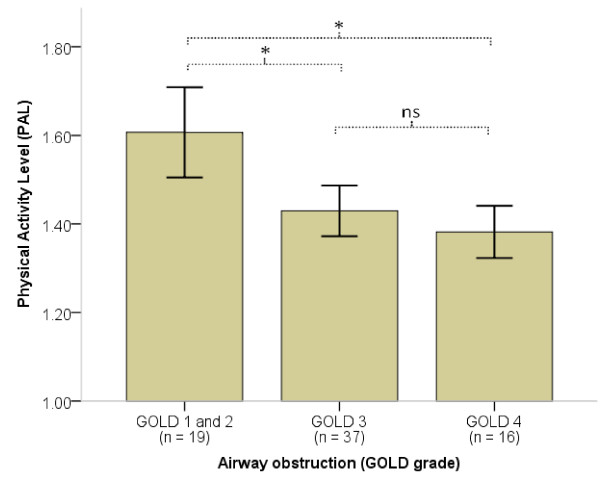
**Physical activity level (PAL) presented across the airway obstruction classification from the Global Initiative for Chronic Obstructive Lung Disease (GOLD).** Bars are the mean PALs (with 95% CI) in respective GOLD grades. * = p < 0.05, ns = p > 0.05.

The mean PAL was 1.47 ± 0.19. There was a negative correlation between PALs and GOLD grades, r_S_ = -0.447 (p < 0.001), indicating less physical activity with worsening airway obstruction. The differences in PAL were significant between GOLD grades 2 and 3 (p < 0.001) but not between GOLD grades 3 and 4 (p = 0.358) (Figure 1). Sixty-six subjects (92%) were *very inactive or sedentary,* four were *active or moderately active* and two subjects were *vigorously active.* Subjects who were more physically active were characterized by better pulmonary function; higher Body Mass Index, FFMI, walking speed and muscle strength; and less dyspnea (Table 1). Forty-nine percent of the sample (27 female, 8 male) was classified as FFM-depleted. Forty-two percent (19 female, 11 male) did not reach normal walking speed. The maximal quadriceps strength was 31.2 ± 11.2 kg, corresponding to 82 ± 25% of predicted values.

### Univariate associations with PAL

**Figure 2:**
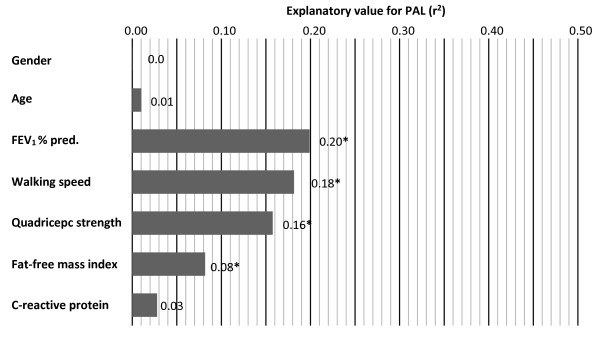
**Figure shows the univariate associations with PAL.** The x-axis represents the explanatory value (*r*^2^) of PAL from the independent variables. FEV_1_ = Forced expiratory volume in one second in percent of predicted,* = p < 0.05.

The explanatory values of FEV_1_, walking speed and quadriceps strength were almost similar (ranging from 16 to 20%) (Figure 2), whereas the correlation for FFMI was considerably lower. No significant associations were identified for age, gender or CRP. Moderate correlations were identified between quadriceps strength and FFMI and between gender and FFMI, whereas all other correlations between independent variables were weak or statistically non-significant (Table 2).

**Table 2:** Correlation matrix of the variables used in the regression model

	**PAL**	**Age**	**Gender**	**FEV**_**1**_	**Walking speed**	**Quadriceps strength**	**FFMI**
PAL							
Age (years)	-0.101						
Gender (female = 1)	-0.012	-0.207					
FEV_1_ (percent predicted)	0.446**	0.075	0.093				
Walking speed (m/s)	0.426**	0.046	-0.172	0.253*			
Quadriceps strength (kg)	0.397**	-0.088	-0.410**	0.120	0.315**		
FFMI (kg/m^2^)	0.286*	0.098	-0.591**	0.089	0.147	0.524**	
CRP (log values)	-0.167	0.139	-0.058	-0.006	-0.038	0.063	0.106

Note: N = 72. FEV_1_ = Forced expiratory volume in one second in percent of predicted, FFMI = fat-free mass index, CRP = C-reactive protein **p < .001, *p < .05 (two-tailed).

### Multivariate model for explanation of PAL

**Figure 3:**
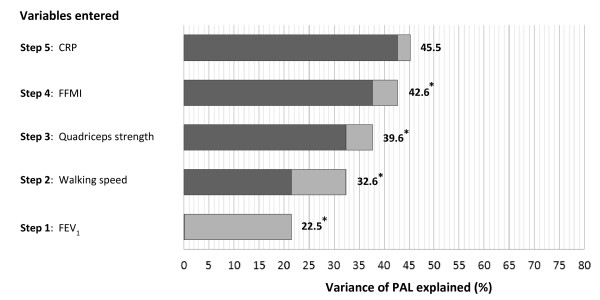
**Graphical presentation of the evolving regression model adjusted for age and gender.** The darker color represents the proportion of variance explained (R^2^) from the previous step, and the light grey represents the R^2^ change from the variable entered at that step. Numbers outside of the bars are the total R^2^ at the current step, * = significant improvement from previous step, CRP = C-reactive protein, FFMI = fat-free mass index, FEV_1_ = Forced expiratory volume in one second in percent of predicted.

In a hierarchical multiple regression analysis, FEV_1_ accounted for 22.5% of the variation in PAL when adjusting for age and gender (Figure 3). Further improvements to the model were gained by the inclusion of self-selected walking speed (10.1%), quadriceps strength (7.0%) and FFMI (3.0%), details of the model is presented in Table 3. Age and gender did not contribute significantly to the model; nor did CRP when adjusting for the previous variables entered. The fit of the final regression model was R^2^ = 0.45, (p < 0.001).

**Table 3:** Independent predictors of physical activity level (PAL)

**Variable**	**PAL**				
	**B**	**SE**	**95% CI**	**Sig.**	***Βeta***
Age (years)	-0.002	0.003	-0.007, 0.003	0.489	-0.054
Gender (female = 1)	0.089	0.048	-0.004, 0.183	0.062	0.221
FEV_1_ (percent predicted)	0.004	0.001	0.001, 0.006	0.002	0.305
Walking speed (m/s)	0.232	0.087	0.062, 0.402	0.008	0.282
Quadriceps strength (kg)	0.004	0.002	0.000, 0.008	0.041	0.242
FFMI (kg/m^2^)	0.021	0.011	0.000, 0.042	0.047	0.240
Log of CRP	-0.032	0.017	-0.066, 0.002	0.065	-0.174

Note: N = 72. FEV_1_ = Forced expiratory volume in one second in percent of predicted, FFMI = fat-free mass index, CRP = C-reactive protein, CI = confidence interval, FEV_1_ = forced expiratory volume in one second.

## Discussion

The new information added from this study is that variations in physical activity can be explained only to a degree by lung function but by adding information from simple tests of walking speed and lower extremity strength, substantial improvements in the explanation of the variability in PAL is possible.

In this study, FEV_1_ explained approximately 20% of the variability in PAL. Previous studies have shown moderate associations between higher GOLD grades and both lower PALs [23] and lower numbers of steps/day [24] and with not performing the recommended amount of physical activity [25]. Walker et al. [26] reported that changes in both overall activity and lower limb activity were related to FEV_1_ and that there were higher levels of leg activity among subjects with better FEV_1_. The mechanism behind this association could be dynamic hyperinflation [27]. The altered mechanical properties of the lungs and airways in COPD reduce expiratory flow and limit the time for expiration, especially during physical exertion, leading to increased end-expiratory lung volume [28]. If increased discomfort is apparent in physical activities, subjects are likely to limit their activity to avoid negative experiences of dyspnea, thereby contributing to a worsening of the condition from the disuse of muscles [29]. This model is supported by our data, given that both dyspnea and lung function worsened as PAL decreased. It is reported that walking distance from a 6-minute walk test (6MWT) is a stronger determinant of daily activity than FEV_1_, self-efficacy and health-status [30].

A novel finding in the present study was that the explanatory value of self-selected walking speed towards PAL was of the same magnitude as the explanatory value of FEV_1_. In this study, walking was measured as walking speed over 30 meters. The 30-meter walk test has been shown to correlate well with the 6MWT [19]. Pitta et al. showed that patients with COPD spent less time walking in daily life than did age-matched controls and also walked at lower walking speeds [31]. Furthermore, the authors concluded that the 6-minute walk distance (6MWD) was the best surrogate marker for inactivity in COPD. Our results are therefore in accordance with the results of previous studies confirming the value of walking performance with regard to daily activity [31] However, to our knowledge, the finding of walking speed as an independent predictor of daily physical activity has been reported in only one previous study [32]. DePew et al. measured walking performance using the 4-meter gait speed test and the 6MWT in a sample of chronic lung disease subjects. In agreement with our present results, the association between walking speed and PAL was modest (*r* 0.32), but DePew et al. did not present a multivariate prediction model for PAL, so further comparison is not possible. In the present study, 42% of the participants had a lower than normal walking speed (< 1.0 m/s). A slow walking phenotype has been described in COPD, characterized by both worse exercise capacity and health status [33], but the impact of slow walking on physical activity levels has not been reported previously. The mechanism behind the reduction in speed with COPD is likely quite complex, but a feasible explanation is that it reflects a global sign of the constraints imposed by the airway obstruction and dyspnea. Lower speed likely results in less oxygen consumption in the muscles and thereby less ventilatory stress and dyspnea. In this scenario, muscular dysfunction must also be considered because it could impact both walking performance and physical activity.

The proportion of variability of PAL explained by quadriceps strength in the present study was almost of the same magnitude as the variability explained by lung function and walking speed. An association between quadriceps strength and daily activity was also reported in the study by Pitta et al. [31]. However, the maximal muscle strength in our sample was well preserved at the group level (82% predicted) compared with the reference values [21], which is somewhat contradictory to the results of the Pitta study. However, it is important to consider that muscular performance might be affected despite preserved maximal strength [34,35]. Coronell et al. found that the endurance of the quadriceps muscles was more impaired than was maximal strength and that impairment was present even in subjects with mild to moderate disease without sedentarism. Borst et al. [35] showed that reduced oxidative phenotype in COPD was related to reduced quadriceps endurance, but not with total activity or its intensity. Lower PALs were not associated with lower quadriceps force in newly diagnosed subjects, as shown by Van Reemortel et al. [36].

Nearly half of the participants were FFM depleted with the least physically active subjects being most affected. FFM depletion was more prevalent among women than men (61% vs. 29%), with a substantially larger gender discrepancy than was described in a Dutch outpatient sample [5]. In the present study, the explanatory value of FFMI towards PAL was relatively low. The reasons may be that we included quadriceps strength in our model and that our subjects had well-preserved quadriceps strength. In our study, there was a relatively strong association between quadriceps strength and FFMI (*r* = .52), which is in accordance with the findings of a previous study [5].

Systemic inflammation has previously been linked to muscle dysfunction in COPD [37] but was not confirmed in our study. Perhaps the large proportion of subjects displaying low FFMI contributed to this finding. Eagan et al. [38] reported that patients with low FFMI did not have increased levels of CRP or soluble TNF-receptor 1; instead, they found the opposite to be true.

### Methodological considerations

We used an activity monitor that operated on the basis of motion sensors, in contrast to the more recent literature, in which accelerometers have been used. We considered the choice of the monitor to be a strength of the study because of its proven validity in measuring energy expenditure in COPD [18]. Furthermore, the sampling method used was not random, which could have introduced selection bias into the study. However, the aim was to include a relatively homogenous sample of COPD subjects by excluding many of the common co-morbid conditions observed in COPD. Other lung function variables than FEV_1_ and forced vital capacity would have been needed in order to assess whether the association between low FEV_1_ and low PAL was related to dynamic hyperinflation or not. The cross-sectional design does not permit any cause-effect relationships to be determined but allows for the generation of new hypotheses. The method for quantifying quadriceps strength was not tested rigorously for validity or reliability, which should be taken into account when interpreting the results. Body composition and FFM depletion were assessed by dual-energy x-ray absorptiometry, a valid method for assessing body composition.

Our findings suggest that incorporating measures of physical function into clinical practice would be of great value. Activity monitoring suffers from being costly and technically challenging. Having simpler measures for identifying subjects who are sedentary would open new possibilities for interventions. It is also important to note that two of the three major correlates of PAL, walking speed [39,40] and muscle strength [41], can be improved by pulmonary rehabilitation. A novel finding is that in a sample not burdened by comorbidity, impairments in walking speed rather than muscle strength seem valuable for identifying the most sedentary subjects. These subjects can be characterized by reduced walking speed, FFM depletion and poor lung function. Because impairments in one of these factors have been shown to impact mortality, the combined impacts of simultaneous impairments in all three factors should likely be of extra concern for clinicians.

## Conclusions

We conclude that apart from lung function, walking speed and muscle strength are important correlates of physical activity. Further explorations of the longitudinal effects of the factors characterizing the most inactive subjects are warranted.

## Abbreviations

COPD: chronic obstructive pulmonary disease; CRP: C-reactive protein; FEV1: forced expiratory volume in one second (L); FFM: fat-free mass (kg); FFMI: fat-free mass index (kg/m^2^); GOLD: Global Initiative for Chronic Obstructive Lung Disease; PAL: physical activity level (total energy expenditure / resting metabolic rate); RMR: resting metabolic rate; TEE: total energy expenditure; 6MWD: 6-minute walking distance (result of a 6-minute walk test).

## Competing interests

The authors declare that they have no competing interests.

## Author contributions

MA: contributed to study design, performed the statistical analysis, contributed to the interpretation of data and drafted the manuscript. FS contributed to study design, to the interpretation and acquisition of data and to the critical review of the manuscript. AMG contributed to the acquisition of data and to the critical review of the manuscript. US contributed to study design, to the acquisition of data and to the critical review of the manuscript. CJ participated in the statistical analysis, the interpretation of data and to the critical review of the manuscript. ME contributed to study design, to the interpretation of data and to the critical review of the manuscript. All authors have read and approved the final version of the manuscript.

## References

[B1] Vestbo J Hurd SS Agustí AG Jones PW Vogelmeier C Anzueto A Barnes PJ Fabbri LM Martinez FJ Nishimura M Stockley RA Sin DD Rodriguez-Roisin R Global strategy for the diagnosis, management, and prevention of chronic obstructive pulmonary disease: GOLD executive summary Am J Respir Crit Care Med 2013 187 347 365 10.1164/rccm.201204-0596PP 22878278

[B2] Watz H Waschki B Boehme C Claussen M Meyer T Magnussen H Extrapulmonary effects of chronic obstructive pulmonary disease on physical activity: a cross-sectional study Am J Respir Crit Care Med 2008 177 743 751 10.1164/rccm.200707-1011OC 18048807

[B3] Barnes PJ Celli BR Systemic manifestations and comorbidities of COPD Eur Respir J 2009 33 1165 1185 10.1183/09031936.00128008 19407051

[B4] Laveneziana P Palange P Physical activity, nutritional status and systemic inflammation in COPD Eur Respir J 2012 40 522 529 10.1183/09031936.00041212 22941542

[B5] Vermeeren MAP Creutzberg EC Schols AMWJ Postma DS Pieters WR Roldaan AC Wouters EFM Prevalence of nutritional depletion in a large out-patient population of patients with COPD Respir Med 2006 100 1349 1355 10.1016/j.rmed.2005.11.023 16412624

[B6] Bossenbroek L de Greef MHG Wempe JB Krijnen WP Ten Hacken NHT Daily physical activity in patients with chronic obstructive pulmonary disease: a systematic review COPD 2011 8 306 319 10.3109/15412555.2011.578601 21728804

[B7] Mador MJ Patel AN Nadler J Effects of pulmonary rehabilitation on activity levels in patients with chronic obstructive pulmonary disease J Cardiopulm Rehabil Prev 2011 31 52 59 10.1097/HCR.0b013e3181ebf2ef 20724933

[B8] Egan C Deering BM Blake C Fullen BM McCormack NM Spruit MA Costello RW Short term and long term effects of pulmonary rehabilitation on physical activity in COPD Respir Med 2012 106 1671 1679 10.1016/j.rmed.2012.08.016 23063203

[B9] Casanova C Cote CG Marin JM de Torres JP Aguirre-Jaime A Mendez R Dordelly L Celli BR The 6-min walking distance: long-term follow up in patients with COPD European Respiratory Journal 2007 29 535 540 10.1183/09031936.00071506 17107991

[B10] Waschki B Kirsten A Holz O Müller K-C Meyer T Watz H Magnussen H Physical activity is the strongest predictor of all-cause mortality in patients with COPD: a prospective cohort study Chest 2011 140 331 342 10.1378/chest.10-2521 21273294

[B11] Garcia-Rio F Rojo B Casitas R Lores V Madero R Romero D Galera R Villasante C Prognostic value of the objective measurement of daily physical activity in patients with COPD Chest 2012 142 338 346 10.1378/chest.11-2014 22281798

[B12] Bauman AE Sallis JF Dzewaltowski DA Owen N Toward a better understanding of the influences on physical activity: The role of determinants, correlates, causal variables, mediators, moderators, and confounders Am J Prev Med 2002 23 2, Supplement 1 5 14 10.1016/S0749-3797(02)00469-5 12133733

[B13] Bauman AE Reis RS Sallis JF Wells JC Loos RJF Martin BW Correlates of physical activity: why are some people physically active and others not? Lancet 2012 380 258 271 10.1016/S0140-6736(12)60735-1 22818938

[B14] Miller MR Hankinson J Brusasco V Burgos F Casaburi R Coates A Crapo R Enright P van der Grinten CP Gustafsson P Jensen R Johnson DC MacIntyre N McKay R Navajas D Pedersen OF Pellegrino R Viegi G Wanger J Standardisation of spirometry Eur Respir J 2005 26 319 338 10.1183/09031936.05.00034805 16055882

[B15] Quanjer PH Tammeling GJ Cotes JE Pedersen OF Peslin R Yernault JC Lung volumes and forced ventilatory flows. Report Working Party Standardization of Lung Function Tests, European Community for Steel and Coal. Official Statement of the European Respiratory Society Eur Respir J Suppl 1993 16 5 40 8499054

[B16] Hustvedt B-E Christophersen A Johnsen LR Tomten H McNeill G Haggarty P Løvø A Description and validation of the ActiReg: a novel instrument to measure physical activity and energy expenditure Br J Nutr 2004 92 1001 1008 10.1079/BJN20041272 15613263

[B17] Human energy requirements: Report of a Joint FAO/WHO/UNU Expert Consultation 2001 Rome

[B18] Arvidsson D Slinde F Nordenson A Larsson S Hulthén L Validity of the ActiReg system in assessing energy requirement in chronic obstructive pulmonary disease patients Clin Nutr 2006 25 68 74 10.1016/j.clnu.2005.09.001 16239051

[B19] Andersson M Moberg L Svantesson U Sundbom A Johansson H Emtner M Measuring walking speed in COPD: test-retest reliability of the 30-metre walk test and comparison with the 6-minute walk test Prim Care Respir J 2011 20 434 440 10.4104/pcrj.2011.00082 21938352 PMC6549884

[B20] Cesari M Kritchevsky SB Penninx BW Nicklas BJ Simonsick EM Newman AB Tylavsky FA Brach JS Satterfield S Bauer DC Visser M Rubin SM Harris TB Pahor M Prognostic value of usual gait speed in well-functioning older people–results from the Health, Aging and Body Composition Study J Am Geriatr Soc 2005 53 1675 1680 10.1111/j.1532-5415.2005.53501.x 16181165

[B21] Seymour J Spruit M Hopkinson N Natanek S Man W Jackson A Gosker H Schols A Moxham J Polkey M Wouters E The prevalence of quadriceps weakness in COPD and the relationship with disease severity Eur Respir J 2010 36 81 88 10.1183/09031936.00104909 19897554 PMC3039205

[B22] Mahler D Wells C Evaluation of clinical methods for rating dyspnea Chest 1988 93 580 586 10.1378/chest.93.3.580 3342669

[B23] Watz H Waschki B Meyer T Magnussen H Physical activity in patients with COPD Eur Respir J 2009 33 262 272 19010994 10.1183/09031936.00024608

[B24] Jehn M Schmidt-Trucksäss A Meyer A Schindler C Tamm M Stolz D Association of daily physical activity volume and intensity with COPD severity Respir Med 2011 105 1846 1852 10.1016/j.rmed.2011.07.003 21803556

[B25] Pitta F Troosters T Probst VS Lucas S Decramer M Gosselink R Potential consequences for stable chronic obstructive pulmonary disease patients who do not get the recommended minimum daily amount of physical activity J Bras Pneumol 2006 32 301 308 10.1590/S1806-37132006000400008 17268729

[B26] Walker PP Burnett A Flavahan PW Calverley PMA Lower limb activity and its determinants in COPD Thorax 2008 63 683 689 10.1136/thx.2007.087130 18487318

[B27] Garcia-Rio F Lores V Mediano O Rojo B Hernanz A López-Collazo E Alvarez-Sala R Daily physical activity in patients with chronic obstructive pulmonary disease is mainly associated with dynamic hyperinflation Am J Respir Crit Care Med 2009 180 506 512 10.1164/rccm.200812-1873OC 19542481

[B28] Thomas M Decramer M O’Donnell DE No room to breathe: the importance of lung hyperinflation in COPD Prim Care Respir J 2013 22 101 111 10.4104/pcrj.2013.00025 23429861 PMC6442765

[B29] Polkey MI Moxham J Attacking the disease spiral in chronic obstructive pulmonary disease Clin Med 2006 6 190 196 10.7861/clinmedicine.6-2-190 16688981 PMC4953207

[B30] Belza B Steele BG Hunziker J Lakshminaryan S Holt L Buchner DM Correlates of physical activity in chronic obstructive pulmonary disease Nurs Res 2001 50 195 202 10.1097/00006199-200107000-00003 11480528

[B31] Pitta F Troosters T Spruit MA Probst VS Decramer M Gosselink R Characteristics of physical activities in daily life in chronic obstructive pulmonary disease Am J Respir Crit Care Med 2005 171 972 977 10.1164/rccm.200407-855OC 15665324

[B32] DePew ZS Karpman C Novotny PJ Benzo RP Correlations between gait speed, six-minute walk, physical activity, and self-efficacy in severe chronic lung disease Respir Care 2013 10.4187/respcare.02471 PMC4005406 23696689

[B33] Kon SS Patel MS Canavan JL Clark AL Jones SE Nolan CM Cullinan P Polkey MI Man WD-C Reliability and validity of the four metre gait speed in COPD Eur Respir J 2012 10.1183/09031936.00162712 23222875

[B34] Coronell C Orozco-Levi M Méndez R Ramírez-Sarmiento A Gáldiz JB Gea J Relevance of assessing quadriceps endurance in patients with COPD Eur Respir J 2004 24 129 136 10.1183/09031936.04.00079603 15293615

[B35] van den Borst B Slot IGM Hellwig VACV Vosse BAH Kelders MCJM Barreiro E Schols AMWJ Gosker HR Loss of quadriceps muscle oxidative phenotype and decreased endurance in patients with mild-to-moderate COPD J Appl Physiol 2013 114 1319 1328 10.1152/japplphysiol.00508.2012 22815389

[B36] Remoortel HV Hornikx M Demeyer H Langer D Burtin C Decramer M Gosselink R Janssens W Troosters T Daily physical activity in subjects with newly diagnosed COPD Thorax 2013 10.1136/thoraxjnl-2013-203534 PMC3786635 23604460

[B37] Decramer M Lacquet LM Fagard R Rogiers P Corticosteroids contribute to muscle weakness in chronic airflow obstruction Am J Respir Crit Care Med 1994 150 11 16 10.1164/ajrccm.150.1.8025735 8025735

[B38] Eagan TML Aukrust P Ueland T Hardie JA Johannessen A Mollnes TE Damås JK Bakke PS Wagner PD Body composition and plasma levels of inflammatory biomarkers in COPD Eur Respir J 2010 36 1027 1033 10.1183/09031936.00194209 20413541

[B39] Evans RA Hill K Dolmage TE Blouin M O’Hoski S Brooks D Goldstein RS Properties of self-paced walking in chronic respiratory disease: A patient goal-oriented assessment Chest 2011 140 737 743 10.1378/chest.10-3104 21393393

[B40] Dolmage TE Evans RA Hill K Blouin M Brooks D Goldstein RS The effect of pulmonary rehabilitation on critical walk speed in patients with COPD: a comparison with self-paced walks Chest 2012 141 413 419 10.1378/chest.11-1059 21778262

[B41] Pitta F Troosters T Probst VS Langer D Decramer M Gosselink R Are patients with COPD more active after pulmonary rehabilitation? Chest 2008 134 273 280 10.1378/chest.07-2655 18403667

